# The Nutritional and Health Effects of the COVID-19 Pandemic on Patients with Diabetes Mellitus

**DOI:** 10.3390/nu12103013

**Published:** 2020-09-30

**Authors:** Monika Grabia, Renata Markiewicz-Żukowska, Anna Puścion-Jakubik, Joanna Bielecka, Patryk Nowakowski, Krystyna Gromkowska-Kępka, Konrad Mielcarek, Katarzyna Socha

**Affiliations:** Department of Bromatology, Medical University of Bialystok, Mickiewicza 2D St., 15-222 Bialystok, Poland; renmar@poczta.onet.pl (R.M.-Ż); anna.puscion-jakubik@umb.edu.pl (A.P.-J.); joanna.bielecka@umb.edu.pl (J.B.); patryk.nowakowski@umb.edu.pl (P.N.); krystyna.gromkowska.kepka@umb.edu.pl (K.G.-K.); konrad.mielcarek@umb.edu.pl (K.M.); katarzyna.socha@umb.edu.pl (K.S.)

**Keywords:** diabetes mellitus, COVID-19, food choices, eating behaviours, lifestyle habits, survey, hygiene, sleep, stress, nutrition

## Abstract

COVID-19 related restrictions aimed at curbing the spread of the coronavirus result in changes in daily routines and physical activity which can have a negative effect on eating and health habits. The aim of the study was to assess the impact of the COVID-19 pandemic on patients with diabetes and their nutrition and health behaviours. A survey conducted in July 2020 included 124 individuals with type 1 (*n* = 90) and 2 (*n* = 34) diabetes mellitus from Poland. To assess nutritional and health behaviours, an online questionnaire covering basic information, anthropometric data, and details regarding physical activity, eating, and hygiene habits was used. Almost 40% of all respondents with type 1 and 2 diabetes mellitus (DM) stated that their disease self-management had significantly improved. Over 60% of all participants declared that they had started eating more nutritious and regular meals during the COVID-19 pandemic. Enhanced hygiene, in particular, during the period, a statistically significant increase in hand sanitiser use was reported by respondents (18% vs. 82%, *p* < 0.001). The study demonstrated that the pandemic had a significant impact on the behaviour of patients with DM. Improved disease self-management and making healthy, informed food and hygiene choices were observed.

## 1. Introduction

Since the emergence of SARS CoV-2, a new coronavirus known as severe acute respiratory syndrome coronavirus-2, at the end of 2019, the related disease called COVID-19 has spread rapidly around the world [1]. From 15 March 2020, a “cordon sanitaire” was formed around Poland and on 20 March 2020 the state of epidemic including home confinement was introduced in the country. At the time of writing, the peak number (599) of daily cases in Poland occurred on 8 June 2020. At the time of commencing the study, there had already been 36,155 infections and 1520 deaths in Poland [2]. From 6 July to 22 July 2020, the period when the survey was conducted, the following regulations were in force in the country: nose-and-mouth coverings in confined public areas; closure of primary and secondary schools, and institutions of higher education; food and drink establishments were operational with enhanced sanitary measures in place (nose-and-mouth coverings required when not at the table, disinfecting tables, keeping a minimum distance of 1.5 m between patrons); gyms and swimming pools were reopened on 6 June 2020; individuals were allowed to socialise indoors in small groups; in most places, access to specialist medical care was provided at outpatient clinics [3]. 

Diabetes mellitus (DM), a metabolic disorder of various etiologies, is characterised by chronic hyperglycaemia and disturbances in insulin secretion or its activity, or both. Type 1 diabetes mellitus (T1DM) is an insulin-dependent, multifactorial autoimmune disease which results in degradation of the beta cells of islets of Langerhans, which causes impaired insulin production and secretion [4]. Patients with T1DM require intensive treatment involving administration of exogenous insulin in the form of multiple daily injections or as continuous subcutaneous infusion of insulin using personal insulin pumps [5]. Type 2 diabetes mellitus (T2DM) is described as a condition of insulin resistance with relative insulin deficiency, commonly caused by qualitative and quantitative secretory defects [4]. Initial treatment of this type of diabetes involves administration of medication to achieve glycaemic stability. Ultimately, many patients require insulin therapy because of progressive failure of the beta cells and development of complications [5]. 

Regulations imposed to curb the transmission of COVID-19 are likely to have an impact on daily routines, including exercise and eating habits. For people with diabetes mellitus, exercise is an integral part of their disease management [6,7]. Patients may also experience increased mental stress caused by the unpredictability of the situation and as a result of social isolation [6]. This may lead to excessive consumption of products rich in simple carbohydrates which can alleviate stress, since their ingestion stimulates the production of serotonin and enhances the mood. Consuming such products is associated with an increased risk of obesity and complications of COVID-19 [8]. A lack of physical activity, poor food choices, and heightened psychological stress may have a detrimental effect on the immune system, which may not produce a proper response when exposed to the new virus [8]. According to a report by the Centers for Disease Control and Prevention, individuals with T1DM and T2DM may be at an increased risk for severe illness from COVID-19 due to susceptibility to lung infection, which is a consequence of DM-related metabolic disturbances and immunosuppression [9,10]. An in vitro study demonstrated that chronic hyperglycaemia changed the innate immune system, thereby acting on chemotaxis, phagocytosis, but also on bactericidal activity of neutrophils and macrophages [11]. According to the American Diabetes Association (ADA), currently available data regarding COVID-19 is not comprehensive enough to show whether individuals with DM, particularly well-controlled DM, are more prone to developing the disease as compared with the general population. However, if patients are not metabolically balanced, they may experience more considerable blood glucose fluctuations which can cause a number of diabetes-related complications. These complications may make patients with DM more susceptible to contracting COVID-19 and other viral infections due to the body’s limited ability to fight them [12]. 

The aim of the present study was to assess the impact of the COVID-19 pandemic on the nutritional and health behaviours of patients with DM. 

## 2. Materials and Methods 

### 2.1. Participants

The study, which collected data via an online survey, was conducted among 124 Polish patients with DM, with a median age of 23 years (lower to upper quartile, 17–35 years old) between 6 July and 22 July 2020 via private Facebook groups of Polish diabetes societies. The main study inclusion criterion was completion of the survey section regarding diabetes, which was a prerequisite for completing the remaining sections. Responses from individuals residing abroad, women with gestational diabetes, and individuals in quarantine were rejected. Each participant was informed of the anonymity and confidentiality of the survey and its purpose. Each respondent was allowed to complete the survey only once and exit it at any time, which would result in unsaved responses. Participants confirmed their voluntary consent for study participation by completing the survey. They could not provide names and personal data. Parents of young children completed the questionnaire on their behalf. The study was conducted in full compliance with national regulations (consent of Bioethical Commission of the Medical University of Bialystok No. R-I-002/587/2019) and the Declaration of Helsinki.

### 2.2. Questionnaire

The questionnaire (see Appendix A) consisted of three sections. The first section contained questions regarding type of diabetes mellitus the participant suffered from, their gender, age, body height and weight, level of education and place of residence. Anthropometric measurements were self-reported. The body mass index (BMI) is a measure used to determine nutritional status. It was calculated using the following formula: body weight in kg divided by height in meters squared. In paediatric patients (under 18 years of age), BMI was interpreted in relation to norms contained in clinical growth charts. The 10th, 85th, and 97th centiles correspond to the limits of underweight, overweight, and obesity, respectively [13]. For adults, the following WHO approved standards were applied: underweight (below 18.5 kg/m^2^), normal (18.5–24.9 kg/m^2^), overweight (25.0–29.9 kg/m^2^) and obese (above 30.0 kg/m^2^) [14]. 

The second section contained questions relating to disease duration, type of treatment received, and result of the HbA1c test performed within three months of questionnaire completion. 

The last section contained questions regarding physical activity, eating behaviours, and hygiene habits such as stress level, daily screen time, and sleep routine. As for physical activity, respondents could indicate whether and how the type of activity they participated in had changed. The activities included dancing, fitness, swimming, running, gym, cycling, gymnastics, and walking. The frequency of exercise sessions could be described by respondents as “I don’t exercise”, “1–2 times per week”, “3–4 times per week”, “5 and more times per week”. Assessment of changes in eating habits was based on the consumption of the following products: coffee, convenience food, dairy products, delivery meals, eggs, energy drinks, fresh bread, fresh fish, fresh vegetables, frozen fish, grain products, homemade bread, nuts, red meat, salty snacks, sweet beverages, sweet snacks, water, and white meat. As for hand washing and sanitiser use, the respondents could indicate the following situations: “after coming home”, “after using the toilet”, “before cooking”, ”after contact with animals”, ”after leaving public transport”, and ”after leaving shops”. The number of hours the respondents spent sleeping fell into the following categories: “under 5–8 h”, “5–8 h”, or “over 8 h”. As for time spent in front of the computer or TV, the respondents could select from the following: “less than 2 h a day”, “2–4 h a day”, “5–7 h a day”, or “8 or more hours a day”. Stress levels could be classified as follows: “low”, “medium”, “high”, and “very high”. 

The questionnaire was based on previously published work of other authors with modifications reflecting the situation under investigation and study cohort [8,15]. Furthermore, questionnaires originally published in foreign languages were translated into Polish and assessed by a Polish native speaker to exclude bias in interpretation. The questionnaire was pretested on a small sample of respondents from the target population to allow for subsequent eradication of formal and substantive errors. 

### 2.3. Statistical Analysis

Statistical analysis was performed using Statistica software (version 13; StatSoft Inc., Krakow, Poland). Normal distribution of the studied variables was checked using the Shapiro–Wilk test. The Mann–Whitney U test was used when data was not symmetrically distributed. Relationships between qualitative features (e.g., periods before and during the COVID-19 pandemic) were evaluated using the Chi-square independence test. In justified cases, Yates’ correction was used. Prior to conducting the survey, a minimum sample size was calculated, which was used for estimating of the number of people who should be tested in order for intended results to be obtained with a specified confidence level (α = 0.95) and a maximum error (10%). The p-value < 0.05 was considered to be statistically significant. Due to the possibility of confounding between variables, additional characteristics of the results for all items in which it occurred were included in the Supplementary Material. The Results section contains outcomes relating to all study participants (adults and children together). Due to the fact that 1/3 of respondents with T1DM were children, we presented the results obtained in this group of patients in a separate subsection. The outcomes obtained, after the exclusion of children under 18 years of age from the study group, were consistent with the results for the entire study cohort. 

## 3. Results

### 3.1. Basic Information 

Characteristics of the study cohort are presented in Table 1. The majority of respondents were individuals with type 1 diabetes mellitus (73%) and women (83%). Among survey participants, 58% of patients with T1DM used personal insulin pumps, while 42% used insulin pens. As for patients with T2DM, 53% used insulin pens and 47% oral drugs. None of the participants reported simultaneous use of insulin injections and oral medication. Among the completed questionnaires, 11% were filled in by parents of children with T1DM. A normal BMI was observed in 59% of the study cohort, overweight in 30%, obesity in 10%, and underweight in 1%. The majority of study participants (57%) had a university degree, 21% of respondents were unemployed, 19% worked in the office, 17% worked from home, and the remainder were students (43%). There were no participants who were quarantined prior to questionnaire completion. Over half (52%) of the respondents lived with their parents, 39% lived with a partner, and the remaining 9% resided alone. 

The study participants were asked if their disease self-management had improved during the COVID-19 pandemic. Among the participants, 46% individuals with T1DM declared that their disease control had deteriorated, 40% stated that it had improved, while 14% did not report any changes.

As for body weight, 39% of study participants reported an increase in body weight during the pandemic, 28% reported an increase of ≤5 kg, and 11% reported >5 kg. Only 31% of patients with DM stated that their body weight did not change. The remaining 30% of patients reduced their body weight, 19% by ≤5 kg and 11% by >5 kg. 

### 3.2. Physical Activity

**Figure 1:**
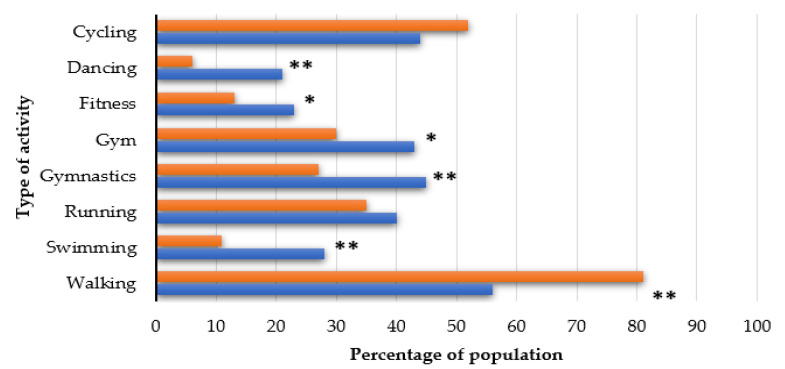
Differences between the type of physical activity “before” and “during” the COVID-19 pandemic. Differences between “before” and “during” the COVID-19 pandemic were evaluated by the Chi-square test (* *p* < 0.05 and ** *p* < 0.01).

Prior to the COVID-19 pandemic, 21% of study participants did not engage in any physical activity, 36% participated in physical activity 1–2 times a week, 31% 3–4 times a week, and 12% over 5 times a week as compared with the period during the pandemic (34%, 41%, 19%, and 6%, respectively). There were statistically (*p* < 0.05) significant differences among the above variables (see Table 2). Figure 1 shows the type of physical activity chosen before and during the COVID-19 pandemic. A statistically significant increase in walking was demonstrated (56% vs. 81%, *p* < 0.001) while a statistically significant decrease in participation in gymnastics, swimming, and dancing (*p* < 0.001 each), gym and fitness classes (*p* < 0.05 each) was observed. Additional characteristics between type of disease and gender are presented in Supplementary Table S1).

### 3.3. Eating Behaviours

When asked if they started eating more healthily during the pandemic, 60% of respondents declared improvements in their dietary habits. Survey results demonstrated that 65% of respondents had started eating more regular meals, in particular main meals. The same total percentage of the study cohort declared that they had started preparing their own meals (see Supplementary Table S2). 

The results revealed that 4% of respondents consumed one to two meals per day before the pandemic as compared with 3% during the pandemic, 54% vs. 50% consumed three to four meals a day, and 42% vs. 47% had more than five meals a day. No statistical significance was demonstrated between the pre-COVID-19 period and the period during the pandemic for any of the above variables, in any of the study participants. More than 30% of respondents admitted that frequency of snacking between meals increased during the pandemic.

**Figure 2:**
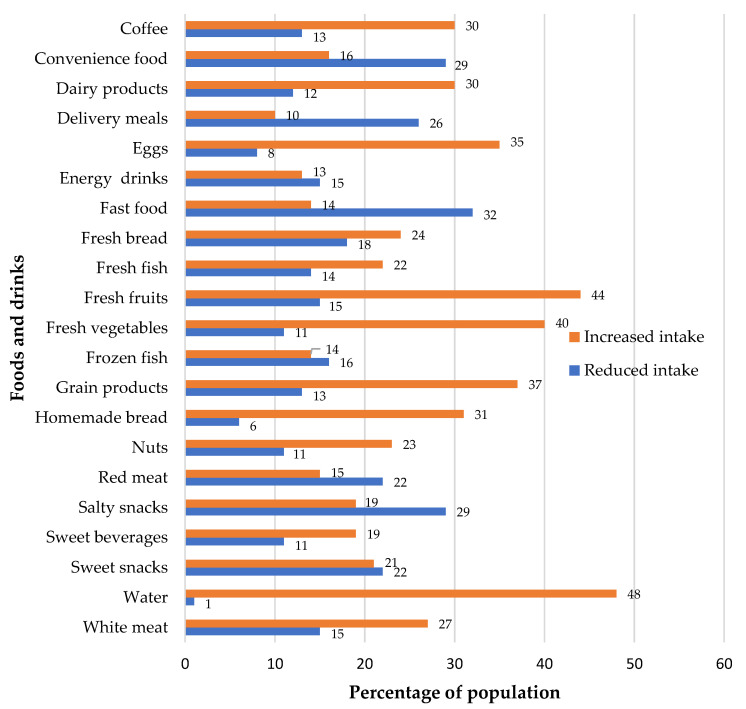
The impact of the COVID-19 pandemic on food and drink intake according to respondents’ responses.

Consumption of selected food products during the COVID-19 pandemic is presented in Figure 2 and the breakdown by the type of diabetes and gender is included in Supplementary Table S3. The most marked increase in intake was revealed for the following products: water (48%), fresh fruit (44%), vegetables (40%), and grain products (37%). The most substantial decrease in consumption was recorded for the following products: fast food (32%), convenience food (29%), salty snacks (29%), delivery meals (26%), red meat (22%), and sweet snacks (22%).

### 3.4. Hygiene Behaviours

The study revealed a statistically significant increase in hand sanitiser use during the COVID-19 pandemic (*p* < 0.01). Prior to the pandemic, 18% of study participants never used hand sanitisers, 64% used them sometimes, while 18% used them very often. Use of sanitising solutions increased during the pandemic, i.e., 89% of respondents declared that they used them very often, while 11% reported using them sometimes. 

**Figure 3:**
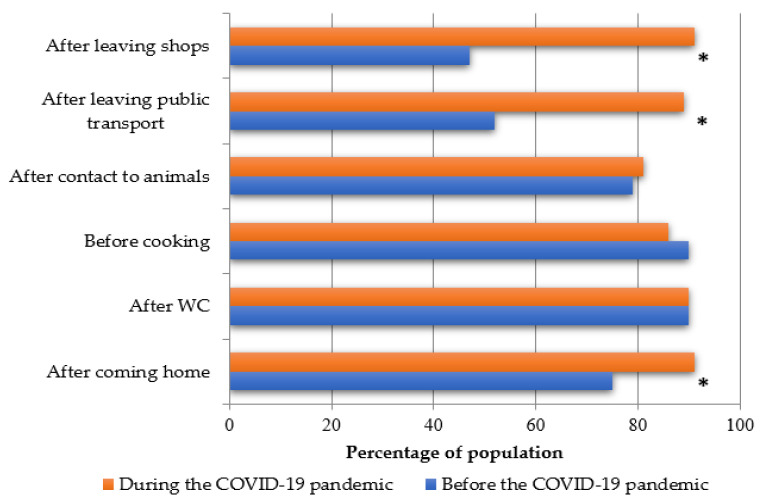
Frequency of hand washing/antibacterial agent use before and during the COVID-19 pandemic. Differences between “before” and “during” the COVID-19 period were evaluated by the Chi-square test (* *p* < 0.001).

A statistically significant relationship between frequent hand washing/antibacterial agent use before and during the COVID-19 pandemic was found (Figure 3). The biggest, statistically significant differences were observed in hand washing after leaving shops (47% vs. 91%, *p* < 0.001), public transport (52% vs. 89%, *p* < 0.001), and after returning home (75% vs. 91%, *p* < 0.001). Additional characteristics regarding the type of disease and gender are presented in Supplementary Table S4).

### 3.5. Lifestyle

A statistically (*p* < 0.001) significant dependence between the period before the start of the COVID-19 pandemic and the time during the pandemic was demonstrated in the number of hours spent in front of the TV or computer. Prior to home confinement caused by COVID-19, only 8% of study participants spent more than 8 h per day in front of the TV or computer, while 27% spent 5–8 h, 43% spent 2–4 h, and the remainder (22%) had less than 2 h of screen time (during the pandemic it was 35%, 43%, 17%, and 5%, respectively). 

The dependence between the period before the COVID-19 pandemic and that during the pandemic, and the number of hours of sleep was also statistically significant (*p* < 0.01), i.e., 17% of respondents declared sleeping more than 8 h per day prior to the pandemic, with the figure reaching 45% during the pandemic. The percentage of study participants sleeping less than the recommended number of hours decreased, i.e., 73% vs. 51% of respondents slept between 5 and 8 h per day, and 10% vs. 4% slept less than 5 h per day. 

**Figure 4:**
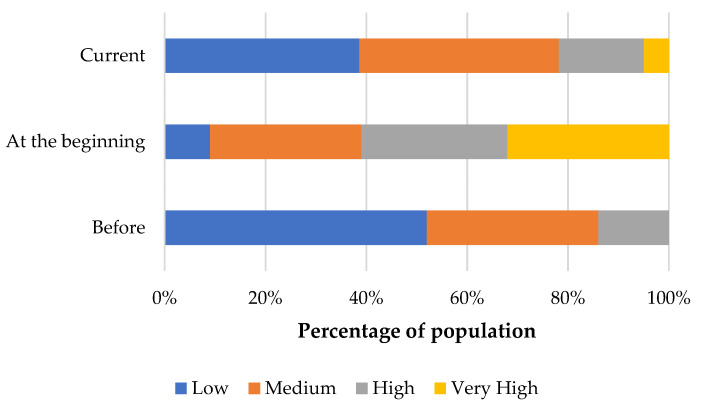
Stress level distribution before, at the beginning of the COVID-19 pandemic, and at the time of questionnaire completion.

Statistically significant (*p* < 0.001) differences were observed in stress levels before, at the beginning of the COVID-19 pandemic, and at the time of completing the questionnaire. Average stress levels increased at the start of the pandemic and returned to nearly pre-pandemic levels at the time of survey completion, as shown in Figure 4. 

Detailed characteristics of the above variables (screen time, sleep routine, and stress levels) in relation to the type of diabetes and gender are presented in Supplementary Tables S5–S7.

### 3.6. Paediatric Outcomes 

In children under 18 years of age (*n* = 35), statistically significant (*p* <0.001) differences were found between the frequency of physical activity before and during the pandemic (see Supplementary Table S8). The percentage of young respondents who practiced gymnastics (57% vs. 37%, *p* < 0.05), swimming (40% vs. 11%, *p* < 0.01), and running (46% vs. 31%, *p* > 0.05) decreased. However, participation in walking (57% vs. 91%, *p* < 0.01) and cycling (51% vs. 63%, *p* > 0.05) increased.

Prior to the pandemic, over 50% of surveyed children consumed one to two meals per day while the remainder had five or more meals. During the COVID-19 pandemic, more than 50% of respondents consumed three to four meals, 40% had five or more meals while fewer than 10% reported having one to two meals per day. Survey results demonstrated a statistically significant relationship between the number of meals consumed per day before the pandemic and the number of meals eaten during the pandemic (*p* < 0.001) (see Supplementary Table S9). 

Study results revealed several improvements in dietary habits of the youngest respondents during the pandemic (see Supplementary Table S10). Over 70% of children reported drinking increased amounts of water during the pandemic, whereas 49% declared that their intake of grain products, fresh bread, fruit, and vegetables had increased. Additionally, 43% of all respondents reported a higher intake of dairy products, while 40% declared increased consumption of eggs. It is worth noting that the intake of fresh fish also grew, i.e., 31% of children reported increased consumption. Additionally, decreased consumption of red meat, fast food, and snacks (both salty and sweet) was observed. 

A statistically significant increase in hand sanitiser use by the youngest respondents during the COVID-19 pandemic (*p* < 0.001) was observed. Prior to the pandemic, 6% of all study participants never used hand sanitisers, 60% used them sometimes, while 34% used them very often. Use of sanitising solutions increased during the pandemic, i.e., 94% of all respondents declared that they used them very often while 6% reported using them sometimes. A statistically significant relationship between frequent hand washing/antibacterial agent use before and during the COVID-19 pandemic was found (see Supplementary Table S11). The biggest statistically significant differences were observed in hand washing after leaving shops (60% vs. 92%, *p* < 0.001) and public transport (69% vs. 86%, *p* < 0.01).

Statistically significant differences were found when screen time of the youngest respondents was analysed (*p* < 0.001. Survey results demonstrated that prior to the pandemic, the majority of young respondents (46%) spent 2–4 h a day in front of the TV or computer, almost one fifth had less than 2 h of screen time a day, whereas only 3% spent 8 or more hours watching TV or using electronic devices. During the COVID-19 pandemic, all respondents declared spending more than 2 h a day in front of the TV or computer, 14% 2–4 h, 60% 5–7 h, and 26% more than 8 h.

There was a statistically significant (*p* < 0.01) increase in the number of hours devoted to sleep, i.e., 74% of children slept for up to 8 h and 26% for more than 8 h per day before the COVID-19 pandemic as opposed to 43% and 57%, respectively, during the pandemic.

Survey results revealed that prior to the pandemic, more than one third of the youngest respondents suffered moderate or high levels of stress. At the start of the pandemic, the majority of participants experienced moderate levels of stress, while over 20% suffered high stress levels. At the time of questionnaire completion, fewer than 10% of all respondents declared suffering very high levels of stress and over 40% of all children evaluated their stress levels as low. Comparison of periods before, at the beginning of the COVID-19 pandemic, and the time of completing the questionnaire revealed statistically significant (*p* < 0.05) relationships (see Supplementary Table S12). 

## 4. Discussion

The present study demonstrated that the pandemic had a significant impact on the nutritional and health behaviour of patients with DM. Frequency of the consumption of both recommended and non-recommended products changed. The COVID-19 pandemic also contributed to more frequent hand washing and increased use of antibacterial agents. 

Due to limited access to medical care, some patients with DM may have experienced difficulty managing their disease. However, the study showed that the majority of respondents were metabolically balanced (median of HbA1c 6.3% to 7.5%). Only 46% felt that they were less able to control their disease. It is worth noting that 40% of all surveyed patients started to monitor their disease more rigorously and over 60% improved their diet by eating more regular, nutrient-dense meals. 

A negative effect of the COVID-19 pandemic and related government-imposed restrictions on movement was limited outdoor activity. As compared with the pre-COVID-19 period, the study demonstrated an increase in the percentage of individuals not practicing any physical activity (21% vs. 34%) and those exercising one to two times per week (36% vs. 41%). The number of people who took up walking increased one and a half times (56% vs. 81%). This can probably be explained by the fact that from 1 April 2020, residents of Poland were allowed to leave home only in the following circumstances: commuting to and from work, voluntary involvement in the fight against the COVID-19 pandemic, and to address matters necessary for everyday living. Outdoor activity was to be kept to a minimum with only walks allowed, as public parks, boulevards and playgrounds were closed. At the time the survey was conducted, the gradual process of relaxing restrictions started. Swimming pools and gyms re-opened a month before the commencement of the study but were not extensively patronised [3]. When the results of our investigation were compared with those of an Italian study by Renzo et al., a similar decrease in the frequency (three to four times per week) of physical activity before and during the lockdown in Italy was noted (27.2% vs. 24.5%, our study 31% vs. 19%). The authors also observed a decline in interest in sports such as fitness classes, running, and gym workout [8]. Nachimuthu et al. published a brief survey conducted among 100 Indian patients with DM which revealed that 80% of respondents monitored their diet regularly and engaged in physical activity at home [16].

The present study revealed that the percentage of individuals consuming five or more meals increased during the COVID-19 pandemic (42% vs. 47%). An increase in the number of meals consumed (7% vs. 19%) was also observed by Ammar et al. who investigated eating habits of healthy people in different countries [15]. The results obtained by Scarmozzino also confirmed that home confinement caused by COVID-19 resulted in increased food consumption for around 50% of respondents [17]. The problem with body weight management experienced by almost 40% of participants of our study may have been caused by a higher number of meals consumed per day, and thus increased calorie intake. A Polish study, conducted during the lockdown, found that individuals with a higher BMI, particularly obese people, were at a heightened risk of adverse dietary changes (increased food consumption and snacking) [18].

A study by Renzo et al. investigated whether there were differences in the consumption of selected products among Italians. As in our study, they observed an increase in the consumption of certain foods such as grain products, hot beverages, eggs, dairy products, fresh bread, white meat, and fresh vegetables [8]. Scarmozzino also observed an increase in consumption of fresh vegetables [17]. Several eating patterns emerged among the youngest participants of our study. On the one hand, over 70% of the surveyed children declared drinking increased amounts of water and nearly half reported that their consumption of grain products, dairy products, fruit, and vegetables had increased. Almost a third of the surveyed children declared a higher consumption of fresh fish. On the other hand, decreased consumption of red meat, fast food, as well as snacks (both salty and sweet) was also reported. White meat and dairy products are beneficial foods which contribute to the prevention of T2DM [19,20]. Increased consumption of fruits and vegetables is associated with a reduced risk of T2DM not only because they contain dietary fibre, essential vitamins and minerals, but also due to the antioxidant and anti-inflammatory effects of their components which include vitamins B and C, carotenoids, and polyphenols [21,22]. 

Similar to the results of our study, a decrease in the consumption of salty snacks, sweet beverages, and delivery food was observed in an Italian survey [8]. A high intake of such products may contribute to unstable diabetes. In patients with DM, a high salt intake may carry a risk of microalbuminuria, particularly in overweight individuals. Sodium retention and blood volume in DM can cause the progression of diabetic microangiopathy [23,24]. Excessive sugar consumption may accelerate the development of T1DM and sugar-sweetened drinks (SSBs) can be particularly harmful to children genetically predisposed to T1DM [24]. High consumption of fructose, SSBs, and high-fructose corn syrup contributes to an epidemic of insulin resistance, visceral obesity, and T2DM [25,26]. Bleich and Wang studied consumption patterns of sugar-sweetened beverages among adult Americans with T2DM. They observed high consumption of these products in young adults and in low-income individuals [26]. 

The glycated haemoglobin (HbA1c) reflects mean glycaemia over the period of approximately three months and is a useful retrospective marker of blood glucose levels as there is an association between blood glucose levels and mean glycaemia, and the risk of developing chronic diabetic complications [27]. A healthy lifestyle involving consumption of regular, nutrient-dense meals prepared at home with quality ingredients brings many beneficial effects, in particular a reduction in HbA1c level. Poor metabolic control is associated with frequent dining out, particularly in fast food establishments, and consumption of high-fat products and snacks between meals [28,29]. 

Our study demonstrated a statistically significant increase in the use of sanitising agents and frequency of hand washing, particularly after coming home and leaving shops or public transport. However, a surprising finding of the study was that the percentage of people sanitising their hands before preparing food decreased. This can be explained by the fact that sanitising solutions are provided at shop entrances and their use is monitored, whereas being home gave individuals a sense of security, and therefore the need for using an antibacterial agent was removed. 

Mental stress activates neuroendocrine processes that affect blood glucose levels by releasing cortisol, endorphins, and growth hormone [30]. This is of adaptive importance for a healthy body but in patients with DM, post-stress hyperglycaemia can exacerbate the disease. Moreover, negative emotions can reduce motivation to adhere to the prescribed treatment and to follow dietary recommendations, which can contribute to poor glycaemic control and increase susceptibility to infections [30,31,32]. The results of our study revealed that at the beginning of the COVID-19 pandemic, stress levels in patients with DM increased sharply (high 14% vs. 29%, very high 0% vs. 32%). At the time the survey was conducted, stress levels were starting to return to pre-pandemic levels. This may have been related to an increase in respondents’ awareness that taking care of their health helps to strengthen the immune system through proper nutrition and compliance with hygiene rules.

Our study has several limitations. The study was retrospective and allowed us to only estimate the impact of the COVID-19 pandemic, some respondents could not fully or accurately recall information they were required to provide. Body weight and height were not measured by a qualified individual, they were self-reported and may not have been accurate. The male subgroup was not sufficiently representative, although it is a common problem in voluntary research. This study was conducted among the inhabitants of one country (Poland). Performing such a study on populations of other countries would allow for a more comprehensive understanding of eating habits and hygiene behaviours of patients with DM. Another limitation was the fact that changes in eating habits were estimated and not correlated with data regarding the sale of particular product groups. The study could only collect data via an online questionnaire since no unauthorised persons were allowed to enter healthcare facilities due to pandemic-related restrictions. The aim of the investigation was to describe eating and health habits that occurred during the COVID-19 pandemic, and therefore these results should not be interpreted in the context of long-term effects. Despite the fact that the sample size was small (*n* = 124), the power of the test reached 91%, with a confidence level (α = 0.95). To our knowledge, this is the first nutritional behaviour study conducted among patients with DM during the COVID-19 pandemic.

## 5. Conclusions

A significant effect of the pandemic on the behaviour of patients with DM was observed. The surveyed patients reported improved disease self-management and making healthy, informed food choices and hygiene habits. The success of nutritional therapy in patients with DM depends on the selection of appropriate food products by individuals, and therefore short reports in the form of questionnaires regarding patients’ nutritional behaviours and their adherence to the recommended dietary regimen should be part of routine nutritional assessment performed by healthcare providers. The results reported in the present study should be used to promote public health during the COVID-19 pandemic. 

## Figures and Tables

**Table 1:** Baseline characteristics of study groups.

Baseline Characteristics of Study Groups	T1DM			T2DM		
	Total (*n* = 90)	Women (*n* = 75)	Men (*n* = 15)	Total (*n* = 34)	Women (*n* = 28)	Men (*n* = 6)
Age (years)	20^A^** (17–28)	20^B^** (17–27)	19 (12–22)	37.0^A^** (31–44)	37.5^B^** (33–45)	31.5 (20–35)
Body weight (kg)	65^A^** (52–75)	63^B^** (52–74)	75 (55–78)	75.0^A^** (66–90)	73.5^B^** (63–90)	78 (70–92)
Height (cm)	168 (162–174)	168 (160–171)	180 (175–182)	168 (160–175)	165 (159–170)	182.5 (179–186)
Body mass index (kg/m^2^)	22.7^A^** (19.3–25.6)	22.0^B^** (19.2–26.3)	22.9 (21.9–23.6)	26.1^A^** (22.4–30.9)	27.0^B^** (23.5–31.2)	23.3 (21.0–25.8)
**Level of education**						
Kindergarten and primary school	17% (15)	14% (11)	27% (4)	-	-	-
Secondary school	34% (31)	36% (27)	27% (4)	21% (7)	10% (3)	67% (4)
University	49% (44)	50% (37)	46% (7)	79% (27)	89% (25)	33% (2)
**Place of residence**						
Village	18% (16)	16% (12)	27% (4)	24% (8)	14% (4)	67% (4)
City (≤150 k inhabitants)	23% (21)	28% (21)	-	32% (11)	39% (11)	-
City (150–250 k inhabitants)	29% (26)	25% (19)	46% (7)	20% (7)	22% (6)	17% (1)
City (≥250 k inhabitants)	30% (27)	31% (23)	27% (4)	24% (8)	25% (7)	17% (1)
Duration of disease^C^**						
Up to 2 years	15% (14)	13% (10)	27% (4)	56% (19)	53% (15)	67% (4)
2–5 years	28% (25)	28% (21)	27% (4)	29% (10)	36% (10)	-
5–10 years	17% (15)	17% (13)	13% (2)	-	-	-
More than 10 years	40% (36)	42% (31)	33% (5)	15% (5)	11% (3)	33% (2)
HbA1c (%) ^D^	6.8 (6.0–7.6)	6.9 (6.2–7.9)	7.5 (6.8–7.9)	6.3 (5.6–8.0)	6.3 (5.7–8.1)	7.5 (7.3–8.6)
Disease self-management^C^*						
No change	17% (15)	20% (15)	-	6% (2)	7% (2)	-
Deteriorated	47% (42)	40% (30)	80% (12)	44% (15)	46.5% (13)	33% (2)
Improved	37% (33)	40% (30)	20% (3)	50% (17)	46.5% (13)	66% (4)
Body mass change^C^**						
No change	28% (25)	20% (16)	60% (9)	41% (14)	43% (11)	33% (2)
Increased ≤5 kg	31% (28)	34% (26)	13% (2)	18% (6)	21% (6)	-
Increased >5 kg	11% (10)	13% (10)	-	12% (4)	7% (2)	33% (2)
Decreased ≤5 kg	23% (21)	26% (17)	27% (4)	9% (3)	11% (3)	-
Decreased >5 kg	7% (6)	7% (6)	-	20% (7)	18% (5)	33% (2)

Values are expressed as median, lower, and upper quartile (Me (Q_1_–Q_3_)) or percentage and number of respondents (% (n)). Abbreviations: Type 1 diabetes mellitus (T1DM) and type 2 diabetes mellitus (T2DM). ^A^ Statistically significant difference between the medians, T1DM vs. T2DM (the Mann–Whitney U test). ^B^ Statistically significant difference between the medians, women with T1DM vs. women with T2DM (the Mann–Whitney U test). ^C^ Statistically significant dependence between variables, T1DM vs. T2DM and women with T1DM vs. women with T2DM (the Chi-square test). ^D^ Results of the glycated haemoglobin (HbA1c) test were collected from 85% of respondents (type of diabetes mellitus: women/men), (T1DM 88%/73% and T2DM 86%/100%). * *p* < 0.05 and ** *p* < 0.001.

**Table 2:** Characteristics of the subgroups, frequency of physical activity before and during the COVID-19 pandemic.

Weekly Activity		Before/During the COVID-19 Pandemic					
	Total * (*n* = 124)		T1DM			T2DM	
		Total * (*n* = 90)	Women * (*n* = 75)	Men (*n* = 15)	Total * (*n* = 34)	Women (*n* = 28)	Men (*n* = 6)
**No activity**	21%/34%	19%/33%	22%/39%	-/7%	26%/35%	25%/35%	33%/33%
1–2 times/week	36%/41%	39%/42%	40%/40%	33%/53%	26%/38%	29%/39%	17%/-
3–4 times/week	31%/19%	30%/18%	27%/17%	47%/20%	35%/24%	39%/22%	17%/33%
≥5 times/week	12%/6%	12%/7%	11%/4%	20%/20%	11%/3%	7%/4%	33%/33%

Abbreviations: Type 1 diabetes mellitus (T1DM), type 2 diabetes mellitus (T2DM). Differences between “before” and “during” the COVID-19 period were evaluated by the Chi-square test (* *p* < 0.05).

## Supplementary Materials

The following are available online at https://www.mdpi.com/2072-6643/12/10/3013/s1, Table S1: Characteristics of the subgroups, type of physical activity before and during the COVID-19 pandemic, Table S2: Characteristics of the subgroups, healthy/regularity of meal consumption during the COVID-19 pandemic, Table S3: Characteristics of the subgroups, variation in food intake during the COVID-19 pandemic, Table S4: Characteristics of the subgroups, frequency of hand washing/antibacterial agent use before and during the COVID-19 pandemic, Table S5: Characteristics of the subgroups, duration of screen time before and during the COVID-19 pandemic, Table S6: Table S6. Characteristics of the subgroups, sleep length before and during the COVID-19 pandemic, Table S7: Characteristics of the subgroups, stress level distribution before, at the beginning of the pandemic and at the time of survey completion, Table S8: Frequency of physical activity before and during the COVID-19 pandemic in children population, Table S9: Number of meals per day in children before and during the COVID-19 pandemic, Table S10: Variation in food intake during the COVID-19 pandemic in children population, Table S11: Frequency of hand washing/antibacterial agent use before and during the COVID-19 pandemic in children population, Table S12: Stress level distribution before, at the beginning of the pandemic and at the time of survey completion in children population.

## Appendix A. Questionnaire

**(Translated)**

**Section 1**

**Q1 What type of diabetes do you have/does your child have?** *I have type 1 diabetes**/** I have type 2 diabetes**/** I am the parent of a child with type 1 diabetes**/** I am the parent of a child with type 2 diabetes* */I have gestational diabetes*

**Q2 Your Gender**: *Female/Male*

**Q3 How old are you? If you are** **the parent, what is your child’s age?**

**Q4 Your** **/your child’s body weight (in kg)**

**Q5 Your** **/your child’s height (in cm)**

**Q6 Has your body weight changed during the pandemic?**

*No/It has increased ≤5 kg/It has increased >5 kg/It has decreased ≤5 kg/It has decreased >5 kg*

**Q7 What stage of education are you at? If you are** **the parent, what stage of education is your child at?** *Kindergarten & Primary school/* *Secondary school/University*

**Q8 Where do you live?** *Village/City* *under 150,000* *inhabitants/City 15–250,000* *inhabitants/City over 250,000* *inhabitants*

**Q9 Are you currently living with someone?** *I live alone**/** I live with my parents**/** I live with my partner*

**Q10 Are you currently working?** *I am studying or learning**/** I’m not working**/** Yes, I* *am working/Yes, I* *am working from home **/** Not applicable*

**Q11 Have you been quarantined?** *No* *, I haven’t**/** Yes, I* *have, the* *coronavirus test result was negative* ***/*** *Yes, I* *have, the* *coronavirus test result was positive*

**Section 2**

**Q12 What type of treatment do you** **receive?** *I use* *insulin pens**/** I use a personal insulin pump**/** I use oral* *anti-diabetic drugs*

**Q13 How long have you had type 1 diabetes?** *under 2 years/2* *–5 years/5* *–10 years/over 10 years*

**Q14 Has your disease** **management changed during the pandemic?** *No,* *it hasn’t **/** Yes, my disease* *self-management has improved **/** Yes,* *my disease* *self-management has declined*

**Q15 What was your most recent glycated haemoglobin (HbA1c) test result? (in %)*** performed within the last three months

*I don’t remember/(value in %)*

**Section 3**

**Q16 Has** **the level of your physical activity changed** **…?**

**Q16.1 …before the pandemic/Q16.2 …during the pandemic**

*I don’t* *exercise/I exercise 1* *–2 times a week/* *I exercise 3* *–4 times a week/* *I exercise 5* *or more times a week*

**Q17 What type of exercise do you do?**

*I don’t do this activity/Before the pandemic/During the pandemic*

- Fitness
- Gymnastics
- Gym
- Running
- Walking
- Swimming
- Cycling
- Dancing

**Q18 How has the amount of sleep you get changed…?**

**Q18.1 …before the pandemic/** **Q18.2 …during the pandemic**

*less 5h/5* *–8h/over 8h*

**Q19 Do you think you started eating better during the pandemic?** *Yes* */No*

**Q20 Has the number of meals you eat changed…?**

**Q20.1 …before the pandemic/Q20.2 …during the pandemic**

*1–2 meals per day/3–4 meals per day/5 or more meals per day*

**Q21 Have you started eating more regular meals (similar hours) during the pandemic?** *No/Yes,* *main meals/Yes, most meals*

**Q22 Have you started cooking more meals yourself?** *No/Yes*

**Q23 Has the frequency of eating certain foods changed during the pandemic?**

*I eat more often**/** I eat less often**/** No change**/** I don’t* *ever eat* *these products*

- Salty snacks (crisps, crackers, bread sticks, etc.)
- Sweet snacks (cakes, cookies, chocolate bars)
- Delivery meals
- Fast Food
- Convenience food
- Red meat (beef, pork)
- White meat (chicken, turkey)
- Fresh fish
- Frozen fish
- Eggs
- Dairy products (milk, yoghurt, cottage cheese)
- Fresh bread
- Homemade bread
- Fresh fruit
- Fresh vegetables
- Grain products (rice, pasta etc.)
- Nuts
- Sweet beverages (except energy drinks)
- Energy drinks
- Coffee
- Water
- Snacks between main meals

**Q24 How often did you use/do you use an antibacterial/sanitising agent?**

**Q24.1 …before the pandemic/Q24.2 …during the pandemic**

*Never**/** Sometimes**/** Often*

**Q25** **Did/do you** **wash your hands or** **use an antibacterial** **/sanitising agent?**

*I do not wash* *my hands or do not use* *an antibacterial/sanitising agent/Before the pandemic* */During the pandemic*

- When you come home
- After using the toilet
- Before preparing food
- After contact with animals
- After leaving the public transport
- After leaving shops

**Q26** **Time spent watching TV** **or using a computer** **/tablet?**

**Q26.1 …before the pandemic/Q26.2 …during the pandemic**

*Less than 2 h* *a day/2–4 h* *a day/5–7 h* *a day/8 h and more* *a day*

**Q27 How do you rate your stress level... ?**

**Q27.1 …before the pandemic/Q27.2 … at the beginning the pandemic/Q27.3 …during the pandemic**

*low/medium/high/very high*
