# Supplementary material for: The Nutritional and Health Effects of the COVID-19 Pandemic on Patients with Diabetes Mellitus
Source: Nutrients. 2020 Sep 30;12(10):3013. doi: 10.3390/nu12103013 (PMC7600117; doi:10.3390/nu12103013)
Supplement: Supplementary file 1 [file nutrients-12-03013-s001.pdf]

nutrients

Supplementary Materials

The Nutritional and Health Effects of the COVID-19 Pandemic on Patients with Diabetes Mellitus

Monika Grabia *, Renata Markiewicz-Żukowska, Anna Puścion-Jakubik, Joanna Bielecka, Patryk Nowakowski, Krystyna Gromkowska-Kępka, Konrad Mielcarek and Katarzyna Socha

Department of Bromatology, Medical University of Bialystok, Mickiewicza 2D St., 15-222 Bialystok, Poland; monika.grabia@umb.edu.pl (M.G.); renmar@poczta.onet.pl (R.M.Ż); anna.puscion-jakubik@umb.edu.pl (A.P.J.); joanna.bielecka@umb.edu.pl (J.B.); patryk.nowakowski@umb.edu.pl (P.N.); krystyna.gromkowska.kepka@gmail.com (K.G.K.), konrad.mielcarek@umb.edu.pl (K.M.); katarzyna.socha@umb.edu.pl (K.S.)

* Correspondence: monika.grabia@umb.edu.pl; Tel.: +48-85-748-5469

Received: 16 August 2020; Accepted: 28 September 2020; Published: date

Table S1. Characteristics of the subgroups, type of physical activity before and during the COVID-19 pandemic.

Type of activity	Before/During the COVID-19 Pandemic					
	T1DM			T2DM		
	Total (n = 90)	Women (n = 75)	Men (n = 15)	Total (n = 34)	Women (n = 28)	Men (n = 6)
Cycling	51%/55%	48%/52%	67%/67%	27%/44%**	14%/43%***	83%/67%
Dancing	19%/8%*	17%/9%	40%/ -	26%/3%***	21%/4%***	50%/ -
Fitness	26%/11%**	26%/23%	27%/13%**	18%/18%	18%/18%	17%/17%
Gym	52%/27%***	53%/27%***	53%/33%***	18%/35%**	17%/50%***	18%/32%
Gymnastic	43%/32%	43%/36%	47%/13%***	50%/12%***	50%/14%***	50%/ -
Running	42%/35%	35%/35%	80%/40%***	36%/36%	25%/32%	83%/50%
Swimming	31%/10%***	28%/12%***	47%/ -	21%/15%	14%/18%	50%/ -
Walking	68%/88%***	55%/81%***	73%/87%**	51%/89%***	50%/89%***	50%/83%

Abbreviations: Type 1 diabetes mellitus (T1DM) and type 2 diabetes mellitus (T2DM). Differences between "before" and "during" the COVID-19 period were evaluated by the Chi-square test (* p < 0.05, ** p < 0.01, *** p < 0.001).

Table S2. Characteristics of the subgroups, healthy/regularity of meal consumption during the COVID-19 pandemic.

Regularity	Total (n = 124)	T1DM			T2DM		
		Total* (n = 90)	Women (n = 75)	Men (n = 15)	Total* (n = 34)	Women (n = 28)	Men (n = 6)
**Healthy eating**							
Yes	40%	40%	39%	47%	38%	43%	17%
No	60%	60%	61%	53%	62%	57%	83%
Regularity of meal consumption*							
No regularity	35%	29%	30%	27%	50%	53%	33%
Main meals only	40%	42%	45%	27%	35%	29%	67%
Most meals	25%	29%	25%	46%	15%	18%	-

Abbreviations: Type 1 diabetes mellitus (T1DM) and type 2 diabetes mellitus (T2DM). * Statistically significant ($p < 0.01$) relationship between total T1DM vs. total T2DM were evaluated by the Chi-square test.

Table S3. Characteristics of the subgroups, variation in food intake during the COVID-19 pandemic.

Products	Increased Intake/Decreased Intake				
	Total (n =124)	T1DM (n = 90)		T2DM (n = 34)	
		Women (n = 75)	Men (n = 15)	Women (n = 28)	Men (n = 6)
**Coffee**	30%/13%	25%/15%	53%/ -	25%/14%	50%/17%
Convenience food	16%/29%	12%/36%	20%/13%	29%/18%	- /33%
Dairy products	30%/12%	31%/11%	40%/13%	22%/11%	33%/33%
Delivery meals	10%/26%	12%/27%	7%/27%	- /33%	7%/22%
Eggs	35%/8%	28%/9%	60%/ -	29%/11%	83%/ -
Energy drinks	13%/15%	16%/19%	7%/ -	7%/7%	17%/50%
Fast food	14%/32%	17%/37%	13%/27%	14%/14%	- /67%
Fresh bread	24%/18%	25%/16%	27%/40%	18%/14%	33%/ -
Fresh fish	22%/14%	19%/13%	53%/ -	18%/18%	- /33%
Fresh fruits	44%/15%	45%/16%	73%/13%	25%/14%	50%/17%
Fresh vegetables	40%/11%	41%/9%	60%/13%	29%/7%	33%/33%
Grain products	37%/13%	40%/16%	40%/13%	29%/7%	33%/ -
Homemade bread	31%/ 6%	25%/5%	73%/7%	22%/11%	33%/ -
Nuts	23%/11%	25%/13%	13%/13%	14%/7%	50%/ -
Red meat	15%/22%	11%/23%	27%/40%	18%/14%	33%/ -
Salty snacks	19%/29%	27%/32%	20%/7%	7%/36%	17%/17%
Water	48%/1%	52%/ -	60%/ -	36%/4%	33%/ -
White meat	27%/15%	27 /12%	60%/7%	11%/29%	33%/ -

Abbreviations: Type 1 diabetes mellitus (T1DM) and type 2 diabetes mellitus (T2DM).

Table S4. Characteristics of the subgroups, frequency of hand washing/antibacterial agent use before and during the COVID-19 pandemic.

	Before/During the COVID-19 Pandemic						
	Total	T1DM			T2DM		
	(*n* = 124)	Total (*n* = 90)	Women (*n* = 75)	Men (*n* = 15)	Total (*n* = 34)	Women (*n* = 28)	Men (*n* = 6)
After leaving shops	47% /91%**	48%/ 90%**	47%/ 91%**	54%/ 87%**	44%/ 94%**	43%/ 93%**	50%/ 100%
After leaving public transport	52%/ 89%**	57%/ 87%**	53%/ 87%**	73%/ 87%*	38%/ 94%**	36%/ 93%**	50%/ 100%
After contact to animals	79%/ 81%	77%/ 81%	75%/ 80%	86%/ 86%	85%/ 82%	89%/ 86%	67%/ 67%
Before cooking	90%/ 86%	90%/ 88%	92%/ 87%	80%/ 93%	91%/ 82%	89%/ 82%	100%/ 83%
After WC	90%/ 90%	91%/ 92%	91%/ 93%	93%/ 87%	88%/ 82%	93%/ 86%	67%/ 67%
After coming home	75%/ 91%*	73%/ 89%*	73%/ 92%*	73%/ 73%	79%/ 87%*	82%/ 97%*	67%/ 100%

Abbreviations: Type 1 diabetes mellitus (T1DM) and type 2 diabetes mellitus (T2DM). Differences between "before" and "during" the COVID-19 period were evaluated by the Chi-square test (* $p < 0.01$ and ** $p < 0.001$).

Table S5. Characteristics of the subgroups, duration of screen time before and during the COVID-19 pandemic.

Duration of activity	Before/During the COVID-19 Pandemic						
	Total*	T1DM			T2DM		
	(*n* = 124)	Total* (*n* = 90)	Women* (*n* = 75)	Men (*n* = 15)	Total* (*n* = 34)	Women* (*n* = 28)	Men (*n* = 6)
< 2 h	22%/5%	20%/6%	17 %/4%	33%/13%	29%/6%	36%/ 7%	- / -
2–4 h	43%/17%	46%/13%	48%/15%	33%/7%	35%/26%	29%/29%	68%/16%
5–7 h	27%/43%	29%/49%	29%/49%	27%/47%	21%/26%	22%/29%	16%/16%
≥8 h	8%/35%	5%/32%	6%/32%	7%/33%	15%/42%	13%/35%	16%/68%

Abbreviations: Type 1 diabetes mellitus (T1DM) and type 2 diabetes mellitus (T2DM). Differences between "before" and "during" the COVID-19 period were evaluated by the Chi-square test (* $p < 0.001$).

Table S6. Characteristics of the subgroups, sleep length before and during the COVID-19 pandemic.

**Sleep length**	**Before/During the COVID-19 Pandemic**						
	**Total* (*n* = 124)**	**T1DM**			**T2DM**		
		**Total** (*n* = 90)**	**Women** (*n* = 75)**	**Men (*n* = 15)**	**Total* (*n* = 34)**	**Women* (*n* = 28)**	**Men (*n* = 6)**
<5 h	10%/4%	12%/2%	15%/3%	- / -	6%/9%	4%/10%	16%/ -
5–8 h	73%/51%	70%/48%	69%/48%	73%/47%	79%/59%	82%/61%	67%/50%
≥8 h	17%/45%	18%/50%	16%/49%	27%/53%	15%/32%	14%/29%	17%/50%

Abbreviations: Type 1 diabetes mellitus (T1DM) and type 2 diabetes mellitus (T2DM). Differences between "before" and "during" the COVID-19 period were evaluated by the Chi-square test (* $p < 0.01$ and ** $p < 0.001$).

Table S7. Characteristics of the subgroups, stress level distribution before, at the beginning of the pandemic and at the time of survey completion.

**Level of stress**	**Total* (*n* = 124)**	**T1DM (*n* = 90)**		**T2DM (*n* = 34)**	
		**Women (*n* = 75)***	**Men (*n* = 15)***	**Women (*n* = 28)**	**Men (*n* = 6)**
**Before the COVID-19 pandemic**					
Low	52%	47%	47%	58%	57%
Medium	34%	36%	53%	18%	33%
High	14%	17%	-	14%	-
Very high	-	-	-	-	-
At the beginning the COVID-19 pandemic					
Low	9%	7%	20%	10%	-
Medium	30%	25%	60%	29%	17%
High	29%	28%	7%	36%	66%
Very high	32%	40%	13%	25%	17%
Currently					
Low	39%	33%	47%	50%	33%
Medium	40%	39%	40%	43%	50%
High	17%	24%	-	7%	17%
Very high	4%	4%	13%	-	-

Abbreviations: Type 1 diabetes mellitus (T1DM) and type 2 diabetes mellitus (T2DM). * Statistically ($p < 0.001$) significant differences were observed in stress levels before, at the beginning of the COVID-19 pandemic and at the time of completing the questionnaire.

Table S8. Frequency of physical activity before and during the COVID-19 pandemic in children population.

**Weekly activity**	**Before/During the COVID-19 Pandemic**	
	**Total (*n* = 35)**	***p*-value**
No activity	12%/26%	0.001
1–2 times/week	34%/43%	
3-4 times/week	34%/26%	
≥5 times/week	20%/5%	

Differences between "before" and "during" the COVID-19 period were evaluated by the Chi-square test.

Table S9. Number of meals per day in children before and during the COVID-19 pandemic.

Number of meals	Before/During the COVID-19 Pandemic	
	**Total (*n* = 35)**	***p*-value**
1–2 meals/day	51%/6%	
3–4 meals/day	- /54%	NS
≥5 meals/day	49%/40%	

Differences between "before" and "during" the COVID-19 period were evaluated by the Chi-square test (NS, non-significant, $p > 0.05$).

Table S10. Variation in food intake during the COVID-19 pandemic in children population.

Products	Increased intake	Decreased intake
Convenience food	23%	40%
Dairy products	43%	20%
Delivery meals	9%	17%
Eggs	40%	6%
Energy drinks	26%	11%
Fast food	20%	29%
Fresh bread	49%	17%
Fresh fish	31%	17%
Fresh fruits	49%	17%
Fresh vegetables	49%	14%
Grain products	49%	23%
Homemade bread	34%	11%
Nuts	20%	14%
Red meat	40%	11%
Salty snacks	23%	26%
Water	71%	-
White meat	29%	14%

Table S11. Frequency of hand washing/antibacterial agent use before and during the COVID-19 pandemic in children population.

	Before/During the COVID-19 Pandemic	
	**Total (*n* = 35)**	***p*-value**
After leaving shops	60%/92%	0.001
After leaving public transport	69%/86%	0.01
After contact to animals	71%/77%	NS
Before cooking	91%/86%	NS
After WC	92%/92%	NS
After coming home	77%/86%	NS

Differences between "before" and "during" the COVID-19 period were evaluated by the Chi-square test (NS, non-significant, $p > 0.05$).

Table S12. Stress level distribution before, at the beginning of the pandemic and at the time of survey completion in children population.

Level of stress	Before	At the beginning	Current	*p*-value
Low	20%	14%	43%	0.02
Medium	34%	46%	37%	
High	34%	17%	14%	
Very high	12%	23%	16%	

Differences between "before", "at the beginning" and "current" the COVID-19 period were evaluated by the Chi-square test.
